# Surgical treatment of ankle fracture with or without deltoid ligament repair: a comparative study

**DOI:** 10.1186/s12891-017-1907-4

**Published:** 2017-12-21

**Authors:** Hong-Mou Zhao, Jun Lu, Feng Zhang, Xiao-Dong Wen, Yi Li, Ding-Jun Hao, Xiao-Jun Liang

**Affiliations:** 1 Department of Foot and Ankle Surgery, Honghui Hospital, Xi’an Jiaotong University College of Medicine, Xi’an, 710054 China; 2 School of Public Health, Xi’an Jiaotong University College of Medicine, Xi’an, 710061 China; 3 Department of Spinal Surgery, Honghui Hospital, Xi’an Jiaotong University College of Medicine, Xi’an, 710054 China

**Keywords:** Ankle fracture, Deltoid ligament, Syndesmosis, Medial clear space

## Abstract

**Background:**

Deltoid ligament (DL) rupture is commonly seen in clinical practice; however the need to explore and surgically repair it is still in debate. The objective of the current study is to compare the outcomes of surgical treatment of ankle fracture with or without DL repair.

**Methods:**

Between 2009 and 2015, Seventy-four ankle fractures with DL rupture were identified and followed. Twenty patients were treated with surgical repair of the DL, while 54 were not. The pre- and post-operative medial clear space (MCS) were measured and the American Orthopaedic Foot and Ankle Society (AOFAS) ankle-hindfoot score and visual analogue scale (VAS) were used for functional evaluation. According to the radiological malreduction of MCS, the odds ratio (OR) and 95% confidence interval (CI) for each potential relative factor were calculated.

**Results:**

The mean followup time was 53.7 months. The mean MCS preoperatively, postoperatively, and at last followup time were 8.7 ± 2.4 (range, 6.2–14.8) mm, 3.7 ± 0.9 (range, 2.6–6.4) mm, 3.6 ± 1.0 (range, 2.6–6.8) mm, respectively. The mean AOFAS score was 86.4 ± 8.1 (range, 52–100) points, and the mean VAS was 1.4 ± 1.4 (range, 0–7) points. During followup, 14.9% (11/74) cases were found to be malreduced (MCS>5 mm), and 5.4% (4/74) went on to failure. Surgical repair of DL can significantly decrease the postoperative MCS (*P*<0.05), and can also decrease the malreduction rate (*P*<0.05). AO/OTA type-C ankle fractures showed a positive correlation with malreduction (OR = 4.38, *P* = 0.03). In this type of injury, surgical repair of the DL can significantly decrease the malreduction rate (*P*<0.05). No significant difference was found between the AO/OTA type-B fracture with or without DL repair.

**Conclusions:**

Surgical repair of the DL is helpful in decreasing the postoperative MCS and malreduction rate, especially for the AO/OTA type-C ankle fractures.

## Background

The deltoid ligament (DL) rupture is highly relevant in clinical practice where ankle injuries are commonly encountered [1–4]. An arthroscopic study reported a partial or total rupture of the deltoid ligament in 39.6% of ankle fracture patients [5]. Another magnetic resonance imaging investigation reported 58.3% of acute ankle fractures have been found with tears of the deltoid ligament [4]. However, in ankle fractures combined with DL rupture, the necessity of surgical repair of the deltoid ligament is always in debate.

Early studies suggested that exploration of the medial side of the ankle and repair of the deltoid ligament were not necessary after anatomical reduction and rigid internal fixation of the lateral malleolus [6–9]. A prospective randomized study reported no difference in early mobilization or in long term results between deltoid ligament repaired and unrepaired groups [9]. However, another study reported that unrepaired deltoid ligament may be a source of persistent pain or pronation deformity when not appropriately treated [10]. Johnson and Hill [11] reported 30 patients with combined fibular fracture and deltoid ligament rupture, where the fibula was fixed and the deltoid ligament was left unrepaired, and the results showed poor symptomatic and functional result in 41% of patients. Until now, the dilemma of whether the deltoid ligament should be surgically repaired in acute ankle fracture is still controversial. Thus, we retrospectively studied the ankle fracture patients with DL rupture in our center to evaluate the need for surgical repair of the deltoid ligament.

## Methods

The current study was approved by the research board in our hospital. The authors retrospectively studied the clinical and radiological outcomes of operative treatment of ankle fractures with DL rupture between March 2009 and December 2015. The inclusion criteria contained: (1) adults greater than 18 years old; (2) with acute closed ankle fractures treated operatively; (3) with preoperative medial clear space (MCS) ≥ 6 mm in anterior-posterior ankle X-rays; (4) and at least 12 months followup. The exclusion criteria contained: (1) the time of injury to surgical intervention more than 14 days; (2) open ankle fractures; (3) DL rupture combined with medial malleolar fracture; (4) pathological fractures; (5) with preoperative dysfunction of the lower limb.

**Fig. 1:**
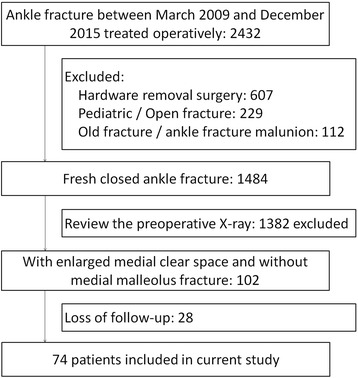
The flowchart of the patients’ selection

A total of 2432 ankle fractures treated operatively were identified initially. According to the inclusion and exclusion criteria, seventy-four patients with 52 males and 22 females were included in current study (Fig. 1). The average age was 39.5 ± 15.5 (range, 18–76) years. Causes of fracture included 42 sprains, 13 falls from height, 12 traffic injuries and 7 sports injuries. According to the AO/OTA classification system [12], 49 type-B and 25 type-C were included; according to Lauge-Hansen classification system [13], there were 49 supination-external rotation (SER), 19 pronation-external rotation (PER) and 6 pronation-abduction (PA) injuries. The preoperative MCS was 8.7 ± 2.4 (range, 6.2–14.8) mm. Twenty patients were treated with surgical repair of DL, and 54 patients were not. The basic information in two groups was similar (Table 1).

**Table 1:** Basic information and functional outcomes between deltoid ligament repaired and unrepaired patients

	DL repaired (*n* = 20)	DL unrepaired (*n* = 54)	*P*-value
Gender (M/F)	16/4	36/18	0.39
Side (L/R)	12/8	30/24	0.80
Causes of injury			
Sprain	10	32	0.75
Fall from high	4	9	
Traffic injury	3	9	
Sports injury	3	4	
AO (Lauge-Hansen) classification			
Type-B (SER)	12	37	0.49
Type-C (PER/PA)	8	17	
Mean follow-up time	46.9 ± 22.5	56.3 ± 23.9	0.13
MCS (mm)	9.5 ± 1.8	8.4 ± 2.5	0.08
Post-operative MCS (mm)	3.3 ± 0.3	3.8 ± 1.0	0.03
Follow-up MCS (mm)	3.2 ± 0.3	3.8 ± 1.2	0.03
Syndesmosis fixation	9	21	0.63
Malreduction (%)	0 (0)	11 (20.4)	0.03
Failure (%)	0 (0)	4 (7.4)	0.57
AOFAS	88.0 ± 5.8	85.9 ± 8.7	0.32
VAS	1.2 ± 0.8	1.6 ± 1.6	0.29

*M* Male, *F* Female, *L* Left, *R* Right, *SER* Supination-external rotation, *PER* Pronation-external rotation, *PA* Pronation-abduction, *MCS* Medial clear space, *AOFAS* American Orthopaedic Foot and Ankle Society ankle and hindfoot score, *VAS* Visual analogue scale

**Fig. 2:**
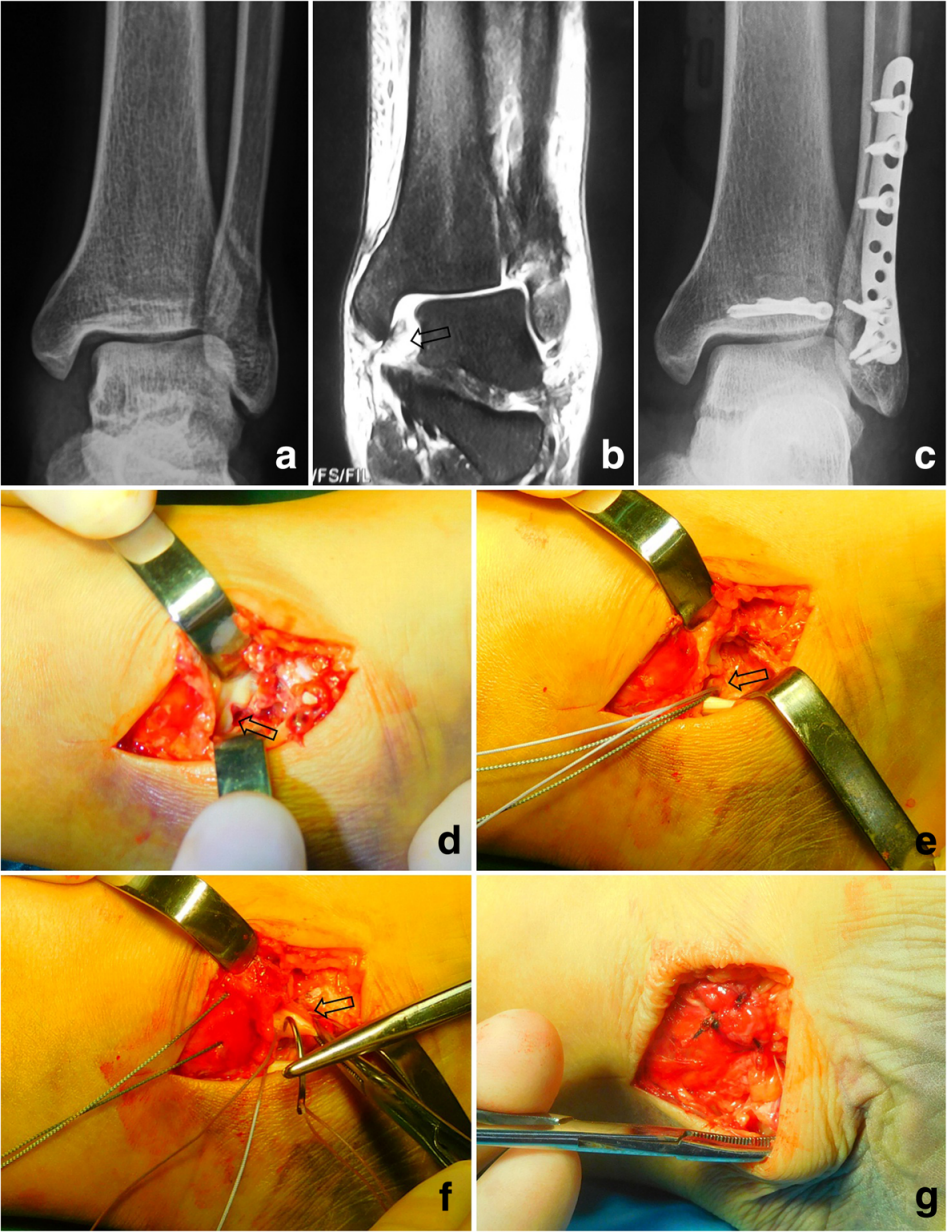
**a** The preoperative X-ray showed enlargement of the medial clear space. **b** MRI revealed the totally rupture of the deep layer of deltoid ligament (arrow). **c** The postoperative X-ray showed good reduction of the medial clear space. **d** Intraoperative photo showed rupture of the deltoid ligament (arrow). **e** A suture anchor was placed in the talus insertion of the deep layer of deltoid ligament (arrow). (**f** and **g**) The deep (arrow) and superficial layers were sutured

All patients were treated with a similar surgical protocol. For the AO/OTA type-B fracture, the fibular length and rotation was restored, and fixed with a small-fragment plate and screws. The posterior malleolar fracture was reduced and fixed for fragments larger than 10% of the articular surface based on the lateral X-ray. If the syndesmotic complex was disrupted, as indicated by its widening during operation, one or two screws were placed across it. For the AO/OTA type-C fracture, the fibula fracture was openly reduced and fixed if it involved the distal two-thirds fragment, but most of the proximal one third fibula fractures were left without fixation after the length and rotation were restored and syndesmotic screws were placed. The posterior malleolar fracture was treated similar to the AO/OTA type-B fracture. For the patients who underwent repair of the DL, reinsertion to the medial malleolus or talus was achieved by suturing directly to the bone, and enhanced with a suture anchor (Fig. 2). The superficial component ruptures were sutured with absorbable suture.

Postoperatively, all patients were immobilized in a short leg cast. At 6 weeks, the cast was taken off, followed by aggressive range of motion and strengthening exercises. The syndesmosis screw was removed in 8 to 12 weeks before full weight-bearing.

### Clinical and radiographic examination

The preoperative, postoperative and final followup anterior-posterior ankle joint X-rays were analyzed. The MCS was measured with Harper’s method [7]. The MCS ≥ 5 mm at any postoperative followup time was defined as malreduction. Treatment failure was defined as symptomatic malreduction and need for any revision surgery.

The American Orthopaedic Foot and Ankle Society (AOFAS) ankle-hindfoot score and visual analogue scale (VAS) was used for functional evaluation at the final followup time [11]. For the failure cases, the AOFAS and VAS scores before revision were included as the final outcomes.

### Statistical analysis

Descriptive statistics were calculated as mean ± standard deviation. Statistical analysis of the included data was performed using Student t test or Pearson chi-square test with the level of significance set at α = 0.05. According to the malreduction rate, odds ratio (OR) and 95% confidence interval (CI) was calculated for the potential relative factor. The statistical analyses were performed with SPSS 17.0 software (SPSS Inc., Chicago, Illinois).

## Results

The mean followup time was 53.7 ± 23.8 (range, 14–97) months. The mean AOFAS at followup time was 86.4 ± 8.1 (range, 52–100) points; and the mean VAS was 1.4 ± 1.4 (range, 0–7) points. The mean postoperative MCS was 3.7 ± 0.9 (range, 2.6–6.4) mm, which was significantly decreased from the preoperative value (*P*<0.01), and maintained at the last followup time (3.6 ± 1.0 (range, 2.6–6.8) mm).

No malreduction or failures occurred in the DL repair group, however, the malreduction rate was 20.4% in unrepair group (*P* = 0.03). The failure rate was 7.4% in the unrepair group, but no significant difference was detected with the numbers available. According to the current study, the mean postoperative MCS was significantly smaller in the DL repair group (*P* = 0.03), and also smaller at the followup time (*P* = 0.03, Table 1). This may be because of the higher malreduced rate in the unrepair group. If the malreducted patients were excluded, the mean MCS decreased to 3.3 ± 0.4 mm postoperatively and 3.2 ± 0.4 mm at final followup time; and the difference disappeared when compared with repair group. No significant difference was detected for AOFAS and VAS scores with the numbers available.

The characteristics of the malreduced patients were summarized in Table 2. Four patients were considered failures and were revised 4–16 months after the initial operation. The other 7 patients all reached good functional outcomes, and painless walking although with increased MCS. The mean AOFAS score of the other 7 patients was 86.6 ± 3.3 (range, 85–95) points, and with a mean VAS score of 1.6 ± 1.1 (range, 0–3) points with a mean follow-up time of 62.6 months. According to our current results, OTA type-C injury was positively correlated with malreduction (Table 3). No correlation was found between malreduction and treatment methods. When compared to the functional outcomes with respect to the OTA classification, the malreduction rate in unrepaired Type-C patients was significantly higher than in unrepaired Type-B patients and repaired Type-C patients (Table 4).

**Table 2:** Characters of malreducted and failure patients

Cases	Gender	Age (y)	Causes of injury	Classification		Fibular fixation	PM fixation	SS fixation	DL repair	FU (m)	AOFAS	VAS	Reversion time (m)	Reversion procedures
				AO	LH									
1	Male	25	Sprain	Type-C	PER-3	Yes	No	No	No	56	85	3		
2	Male	42	Sprain	Type-C	PER-3	No	No	Yes	No	36	88	2		
3	Male	22	Fall	Type-B	SER-4	Yes	No	Yes	No	96	88	2		
4	Male	39	Sprain	Type-B	SER-4	Yes	No	No	No	58	91	1		
5	Male	28	Sport	Type-C	PER-4	Yes	Yes	No	No	86	95	0		
6	Male	18	Sport	Type-B	SER-4	Yes	No	No	No	59	53^a^	7^a^	11	Fibular lengthen, medial debridement and repair
7	Male	47	Traffic	Type-C	PA-3	Yes	No	Yes	No	67	63^a^	6^a^	7	Fibular lengthen, SS fixation, medial debridement and repair
8	Male	52	Traffic	Type-B	SER-4	Yes	Yes	No	No	94	88	1		
9	Male	27	Sport	Type-C	PER-3	Yes	No	Yes	No	76	64^a^	6^a^	16	Fibular lengthen, SS fixation, medial debridement and repair
10	Male	21	Fall	Type-C	PER-4	Yes	No	Yes	No	47	91	1		
11	Female	49	Fall	Type-C	PA-3	Yes	No	No	No	41	63^a^	6^a^	4	SS fixation, medial debridement and repair

^a^The functional score before reversion surgery

*y* Year, *m* Months, *AO* AO classification, *LH* Lauge-Hansen classification, *PM* Posterior malleolus, *SS* Syndesmosis screw, *DL* Deltoid ligament, *FU* Follow-up time, *PER* Pronation-external rotation, *SER* Supination-external rotation, *PA* Pronation-adduction, *AOFAS* American Orthopaedic Foot and Ankle Society ankle and hindfoot score, *VAS* Visual analogue scale

**Table 3:** The correlation of relative factors and malreduction

Relative factors	OR	95% CI	*P*-value
Female gender	0.20	0.02–1.67	0.14
Left side	0.59	0.16–2.12	0.42
Classification			
Type-C	4.38	1.14–16.79	0.03
Treatment			
Fibular fixation	0.50	0.05–5.30	0.56
PM fixation	0.32	0.06–1.59	0.16
SS fixation	0.87	0.23–3.28	0.84
DL repair	0.09	0.01–1.64	0.10

*OR* odds ratio, *CI* confidence interval, *PM* Posterior malleolus, *SS* Syndesmosis screw, *DL* Deltoid ligament

**Table 4:** Outcomes of patients with and without deltoid ligament repair according to different AO classification

	DL repaired (*n* = 20)		DL unrepaired (*n* = 54)	
	Type-B (*n* = 12)	Type-C (*n* = 8)	Type-B (*n* = 37)	Type-C (*n* = 17)
MCS (mm)	9.7 ± 1.6	9.4 ± 1.8	8.4 ± 2.6	8.4 ± 2.5
Post-operative MCS (mm)	3.3 ± 0.3	3.3 ± 0.3	3.6 ± 1.0	4.1 ± 1.1
Follow-up MCS (mm)	3.2 ± 0.3	3.2 ± 0.4	3.5 ± 1.0	4.1 ± 1.2
Malreduction (%)	0 (0)	0 (0)^#^	4 (10.8)^*^	7 (41.2)^*#^
Failure (%)	0 (0)	0 (0)	1 (2.7)	3 (17.6)
AOFAS	86.8 ± 4.8	89.8 ± 7.4	86.3 ± 7.5	84.9 ± 11.1
VAS	1.3 ± 0.6	1.0 ± 1.1	1.4 ± 1.3	2.1 ± 2.2

*MCS* Medial clear space, *AOFAS* American Orthopaedic Foot and Ankle Society ankle and hindfoot score, *VAS* Visual analogue scale

^*^ *P*<0.05. ^#^ *P*<0.05

## Discussion

DL is a complex ligament structure spanning from the medial malleolus to the navicular, talus, and calcaneus bones, and it plays a role in limiting the anterior and posterior translation of the talus and restrains talar abduction. DL repair is performed more frequently than expected, particularly in Weber type B fractures [5]. Surgical treatment of intraarticular fractures is well-accepted as malreduction of the articular surface may cause post-traumatic osteoarthritis rapidly. However, the need for surgical repair of the ruptured DL after the anatomic reduction of the bony structures is still under debate.

Early studies showed that reconstruction of a ruptured DL was not necessary. Harper [7] reported 36 patients, all without repair of DL, and the results show no morbidity or evidence of ligamentous instability. Stromsoe et al. [9] reported a prospective randomized study including 50 patients, where the results showed no difference was found between groups. Baird et al. [6] reported 24 ankle fracture patients with DL rupture, with 21 patients without repair of the DL reaching a good to excellent rate of 90%; however, of the 3 patients with DL repair, 2 had poor results. So, the author concluded that exploration of the medial side of the ankle and repair of the DL are not necessary unless reduction of the lateral malleolus fails to reduce the talus within the ankle mortise. However, Zeegers and van der Werken [8] reported 28 patients without repair of the DL, and 8 (28.6%) had poor results. Johnson and Hill [11] reported 30 patients with combined fibula fracture and DL rupture, where the fibula was fixed and DL was left unrepaired, and the results showed poor symptomatic and functional result in 41% of patients. Tejwani et al. [14] reported that the functional outcome for those with a bimalleolar fracture is worse than that for those with a lateral malleolar fracture and disruption of the DL. In our current study, the functional outcomes between the DL repaired and unrepaired patients reached no significant difference with the numbers available. However, the malreduction rate was significantly higher in DL unrepaired group (0% versus 20.4%). And, in the malreducted patients, 36% (4/11) failed and required revision; although the other 64% (7/11) with increased posterior MCS reached good functional outcomes with a mean 5 years followup.

**Fig. 3:**
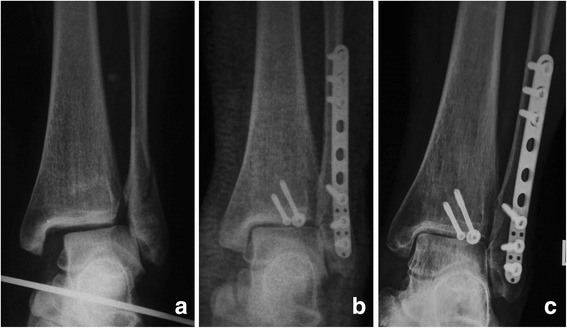
**a** The preoperative X-ray showed an AO/OTA type-B ankle fracture. **b** The patient was treated with open reduction and internal fixation of lateral and posterior malleolus, and the medial clear space was back to normal without surgical repair of the deltoid ligament. **c** Two years followup show good reduction of the medial clear space

**Fig. 4:**
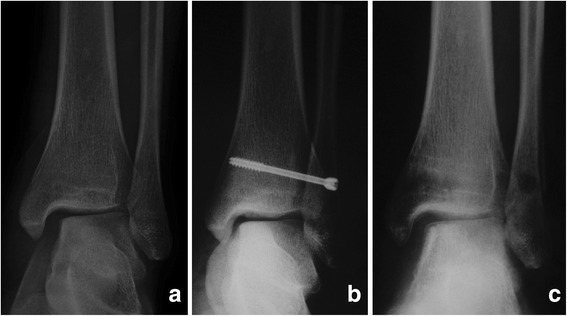
**a** An AO/OTA type-C ankle fracture with enlarged medial clear space and syndesmotic space. **b** The patient was fixed with a syndesmotic screw, and the medial clear space was reduced to normal. **c** One year postoperative X-ray showed malreduction of the medial clear space although without symptoms

For the Weber type-B (SER-4) ankle fracture with DL rupture combined with syndesmosis instability, the use of a syndesmosis screw for temporary fixation was showed to increase the functional outcomes while without DL repair [15]. In our current study, we included 49 Weber type-B patients with DL rupture, and 17 with syndesmosis fixation, and 1 (5.9%) with malreduction of medial malleolar space but with good functional outcomes and without pain. According to our current results, the functional outcomes and radiological outcomes for the Weber type-B patients with DL rupture reached no significant difference with or without DL repair (Table 4). The Weber type-C fractures showed a positive correlation with malreduction in our current study (OR = 5.53, Table 3). However, if the DL was repaired, the malreduction rate decreased significantly even in Weber type-C fracture patients (*P* = 0.04). Lee et al. [16] reported that in the case of high-grade unstable fractures of the lateral malleolus, repair of the anterior DL was adequate for restoring medial stability. We do agree with Hintermann et al. [10] that careful reconstruction of the medial ligaments of the ankle is needed if restoration of full mechanical stability is not proven after internal fixation of Weber type-C ankle fracture. Many authors agreed that after anatomical reconstruction of the lateral malleolus with congruity of the ankle mortise there is no need to explore and repair the ruptured DL [7, 8, 17]. According to our current results, for the Weber type-B ankle fractures, DL repair may be not a necessary procedure after anatomic reduction of the bony structures (Fig. 3, Table 4); however, not for the type-C fractures (Fig. 4, Table 4).

Limitations of our current study included that we used MCS ≥ 6 mm in anterior-posterior ankle X-ray without stress or gravity-stress, which may have a lower sensitivity, although most authors used MCS ≥ 5 mm on the initial unstressed anterior-posterior X-ray to define the DL rupture [7, 18, 19]. Park et al. [19] showed that measurement of an MCS ≥ 5 mm on stress radiographs taken in dorsiflexion-external rotation yielded a sensitivity of 100% (95% CI, 61–100%) and specificity of 100% (95% CI, 89–100%) in cadaveric study. Schuberth et al. [20] reported at an MCS ≥ 5 mm, the false-positive rate for deltoid rupture diminished to 26.9%; and with an MCS ≥ 6 mm, the false-positive rate for deltoid rupture was only 7.7%. As expected, larger MCS thresholds usually resulted in higher specificity but lower sensitivity [21]. Our current method ensured a high specificity for diagnosis. The low sensitivity also explained why we have a smaller percentage of medial ligament injury (6.9%) compared with the previous reports (10–22.6%) [8, 14]. For the postoperative evaluation, we used MCS ≥ 5 mm to define the malreduction just in order to increase the sensitivity. The other limitation was our retrospective design, and not a randomized assignment of the groups. However, the baselines of the two groups were similar, and our results showed very useful information for clinical practice which have not been reported before.

## Conclusions

According to the current study, we concluded that the surgical repair of the DL is helpful in decreasing the postoperative MCS and malreduction rate; especially for the Weber type C ankle fractures. However, the relationship between increased MCS and failure is still unclear. A lot of the patients with increased MCS in the current study still with satisfactory outcomes during long term followup. According to the results, well designed prospective comparative studies focus on the necessary for surgical repair of DL are still needed.

## Abbreviations

AOFAS: American Orthopaedic Foot and Ankle Society
CI: Confidence interval
DL: Deltoid ligament
MCS: Medial clear space
OR: Odds ratio
PA: Pronation-abduction
PER: Pronation-external rotation
SD: Standard deviation
SER: Supination-external rotation
VAS: Visual analogue scale
